# Bilingual comparison of Mandarin and English cognitive bias tasks

**DOI:** 10.3758/s13428-017-0871-0

**Published:** 2017-03-13

**Authors:** Louise Smith, Wing Gi Leung, Bryony Crane, Brian Parkinson, Timothea Toulopoulou, Jenny Yiend

**Affiliations:** 1 Institute of Psychiatry, Psychology and Neuroscience, King’s College London, 16 De Crespigny Park, London, SE5 8AF UK; 2 Department of Experimental Psychology, University of Oxford, Tinbergen Building, 9 South Parks Road, Oxford, OX1 3UD UK; 3 Department of Psychology, University of Hong Kong, Pokfulam Road, Pokfulam, Hong Kong

**Keywords:** Cognitive bias, Attention, Interpretation, Cross-cultural, Information processing, Emotion

## Abstract

Most research into cognitive biases has used Western samples, despite potential East-West socio-cultural differences. One reason is the lack of appropriate measures for non-Westerners. This study is about cross-linguistic equivalence which needs to be established before assessing cross-cultural differences in future research. We developed parallel Mandarin and English measures of interpretation bias and attention bias using back-translation and decentering procedures. We assessed task equivalence by administering both sets of measures to 47 bilingual Mandarin-English speakers. Interpretation bias measurement was similar and reliable across language versions, confirming suitability of the Mandarin versions for future cross-cultural research. By contrast, scores on attention bias tasks did not intercorrelate reliably, suggesting that nonverbal stimuli such as pictures or facial expressions of emotion might present better prospects for cross-cultural comparison. The development of the first set of equivalent measures of interpretation bias in an Eastern language paves the way for future research investigating East-West differences in biased cognition.

Cognitive biases occur when one type of information is consistently favored for further processing over others (Mathews & MacLeod, 2005; Savulich, Freeman, Shergill, & Yiend, 2015) and can occur at any stage of information processing, involving mechanisms including perception, attention, interpretation, and reasoning. Individuals suffering from psychological disorders as well as healthy individuals with heightened vulnerability to psychopathology are characterized by cognitive biases. People tend to demonstrate a characteristic “fingerprint” of cognitive biases specific to their disorder or vulnerability (Yiend, 2010), usually involving attentional prioritization of negative information (Mathews, Ridgeway, & Williamson, 1996) and favoring negative interpretations of emotionally ambiguous information (Eysenck, Mogg, May, Richards, & Mathews, 1991). Conversely, individuals with low vulnerability to psychopathology typically show a bias towards positive information (Hirsch & Mathews, 2000). Evidence from bias manipulation studies shows that biases are important etiological factors contributing to emotional well-being (e.g., Holmes, Lang, & Shah, 2009; Lester, Mathews, Davidson, Burgess, & Yiend, 2011). However, the majority of work has been carried out using Western cohorts (Quinones-Vidal, Lopez-Garcia, Penaranda-Ortega, & Tortosa-Gil, 2004) and there is a surprising dearth of literature from other cultures.

With increasing globalization there is a progressively greater need to understand the cognitive differences between cultures and corresponding implications for well-being, vulnerability to psychopathology, and effective treatment of mental health problems. Between 1990 and 2013, the number of international migrants increased globally by over 50%, bringing the worldwide total to 232 million people (~3% global population; United Nations. General Assembly, 2014). According to 2011 census data, 393,141 (0.70%) people resident in England and Wales stated their ethnic group as Asian or Asian British-Chinese and 387,829 (0.72%) spoke an East Asian language as their main language, of which 22,025 (5.7%) spoke Mandarin (Office for National Statistics, 2011).

Culture, through its institutions and social practices, has the power to shape the norms, values, and ideals held by a population (Sedikedes, Gaertner, & Toguchi, 2003). This idea is manifested in the different ways that Eastern and Western cultures operate; with “collectivist” East Asian cultures typically putting the good of the group before the good of the self, and “individualist” Western cultures typically putting the good of the self before the good of the group (Hui & Triandis, 1986). In their landmark paper, Markus and Kitayama (1991) described how people from individualist cultures tend to make independent self-construals, conceiving of themselves as distinct, autonomous entities. By contrast, people from collectivist cultures tend to make interdependent self-construals, and their relationships with in-group members play a significant role in defining who they are. Likewise, members of individualist cultures conceptualize the self as being relatively stable and unchanging across situations, whereas collectivist cultures see the individual’s relationships and roles as taking priority over distinctive personal attributes (Heine, 2001).

Against this background, it would not be surprising if Easterners showed attenuated positive cognitive biases compared to Westerners, potentially making them more susceptible to negative biases. Indeed, Heine, Lehman, Markus, and Kitayama (1999) reported that the distribution of self-esteem scores was positively skewed above the scale’s theoretical midpoint for Western participants but non-skewed for Eastern participants. Similarly, cross-cultural research into optimistic and pessimistic bias shows that while Westerners tend to be optimistic in the prediction of both negative and positive future events, Easterners were pessimistic in the prediction of negative future events (Chang & Asakawa, 2003). More generally, researchers have argued that the tendency to self-enhance (i.e., to selectively collect and process information that supports a positive self-concept) is substantially stronger in Western than Eastern cultures (Heine, 2005; Heine et al., 1999), or at least that it focuses on different aspects of the self (Sedikides et al., 2003; Sedikides, Gaertner, & Vevea, 2005). In fact, individuals from interdependent cultures may often adopt self-critical rather than self-enhancing strategies in order to achieve genuine rather than perceived self-improvement (Kitayama, Markus, Matsumoto, & Norasakkunkit, 1997). If so, we might expect greater levels of negative bias in Easterners than in Westerners. In support of such a conclusion, research from the regulatory focus perspective shows that Eastern participants attend more to avoidance-oriented information, whereas Western participants attend more to approach-oriented information (Hamamura, Meijer, Heine, Kamaya, & Hori, 2009; Uskul, Sherman, & Fitzgibbon, 2009).

To our knowledge, none of the previous research into cross-cultural differences has investigated the specific cognitive biases that are the focus of this paper. However, consistent with the above suggestions, existing evidence does show that self-construals associated with cultural differences relate to affective well-being. Anxiety has been found to be positively correlated with measures of interdependence (higher levels of anxiety in individuals with higher interdependent self-construal; Rapee & Spence, 2004), but negatively correlated with measures of independence (lower levels of anxiety in those with higher independent self-construals; Hardin, Varghese, Tran & Carlson, 2006; Kim, Kasser, & Lee, 2003; Xie, Leong, & Feng, 2008). Similarly, vulnerability to depression correlates positively with interdependent self-construals and negatively with independent self-construals (Norasakkunkit & Kalick, 2002). Given that Easterners tend to score higher on interdependence and lower on independence than Westerners, these findings again suggest that Easterners may be relatively more susceptible to negative cognitive biases.

The unavailability of appropriate measures has been one limiting factor in the investigation of cross-cultural cognitive biases. In this paper, we report the development of parallel Mandarin and English cognitive bias tasks as a first step in the process of filling this gap in the literature. It is important to ascertain whether Eastern cohorts exhibit the same patterns of cognitive biases in relation to psychopathology as do Western cohorts. Evidence relating to this issue will not only facilitate understanding of cross-cultural cognitive phenomena, but also help to ensure the most effective forms of psychotherapy are developed for Eastern cultures. However, before these cognitive biases can be assessed, it is necessary to develop culturally appropriate measures. It is especially important that materials employing emotional stimuli are presented to individuals in their native language given the evidence that the language people learn first often carries stronger emotional connotations than languages learned subsequently (Altarriba, 2008; Pavlenko, 2008).

Two of the most important cognitive biases relate to attention and interpretation (Yiend & Mathews, 2004). Negative attention bias occurs when attention is systematically captured by an emotionally threatening stimulus over other stimuli types (e.g., neutral stimuli; MacLeod et al., 1986), whereas interpretation bias occurs when ambiguous stimuli are consistently interpreted as being negative in content (Amir, Foa, & Coles, 1998; Eysenck et al., 1991; Mathews & Mackintosh, 2000). The study reported here developed parallel English and Mandarin measures of attention bias, using the emotional Stroop and attention probe tasks, and interpretation bias, using the scrambled sentences (SST) and similarity ratings tasks (SRT). The emotional Stroop and attention probe tasks were chosen due to their widespread use in the measurement of attention bias. Similarly, our measures of interpretation bias were chosen as widely-used measures which have been successfully used to show an association between interpretation bias and vulnerability to psychopathology (Savulich et al., 2015). Of the four tasks used in this study, only the emotional Stroop has been used in cohorts speaking languages other than English, with Spanish, Finnish, and Thai translations reported (Eilola, Havelka, & Sharma, 2007; Sutton, Altarriba, Gianico, & Basnight-Brown, 2007; Winskel, 2013). Comparable performance in Spanish and English, and Finnish and English versions of the emotional Stroop task was found in cohorts of Spanish-English and Finnish-English bilinguals, respectively (Eilola et al., 2007; Sutton et al., 2007). However, all of these comparisons involve Western languages.

Here we chose to develop parallel Mandarin and English versions of the emotional Stroop, attention probe, SST and SRT, by translating the English version into Mandarin and checking the adequacy of the translation by back-translating into English, and using decentering (adjustment of each language form; Werner & Campbell, 1970) until equivalence was achieved. Tasks were then tested using bilingual Mandarin-English speakers who completed each cognitive bias task in both languages to provide psychometric data on the parallel versions.^1^ We predicted that if tasks were valid and if cognitive biases were manifesting similarly in both the native and non-native language, then individuals would show closely correlated bias scores across both Mandarin and English versions.

## Method

### Participants

Participants were eligible for the study if they were aged 16–65 years, spoke fluent Mandarin and English, had no current major physical illness or psychological disorder, and were receiving no psychological therapy or medication for psychological conditions. Ethical approval for the study was granted by King’s College London (KCL) College Research Ethics Committees – Psychiatry, Nursing and Midwifery review committee (REC Reference Number: PNM/13/14-74). Participants were recruited using email circulars to KCL staff and students and flyers around campuses, relevant societies and language schools in central London. To ensure adequate fluency, previously developed language comprehension tests were administered in both languages.^2^ The Mandarin test consisted of 40 multiple-choice questions in which the participant had to choose the character that would complete the sentence correctly. In the English language test, participants had to complete five multiple-choice questions, each one after having read a different paragraph, choosing the option that summarized the preceding paragraph most clearly. Participants were compensated at a flat rate of £20 (~US$30) for their time and any travel expenses they may have incurred.

A total of 56 people completed the study, 43 of them females (76.8%). Duration of residence in the UK ranged from 1 to 131 months (*M* = 30.63 months, *SD* = 34.76). Participants ranged in age from 19 to 41 years, with a mean age of 24.32 years (*SD* = 3.87). All participants had normal or corrected-to-normal vision. All participants passed the Mandarin comprehension task, but nine failed the English language comprehension task and their data were therefore excluded from the analysis. Consequently, the results of the remaining 47 participants are reported. One participant was dichromatic (blue-green colorblind); results for this participant were excluded from the emotional Stroop analysis.

### Design

All participants completed each cognitive bias task in both languages (eight tasks per participant in total) in counterbalanced order. Attention bias tasks included a factor “emotion type” with four levels: physically threatening, socially threatening, positive, and neutral. Interpretation bias tasks include no such factor as they are designed such that presented ambiguous stimuli are interpreted either in a positive or negative fashion and corresponding bias scores calculated (i.e., proportion of positive or negative interpretations made).

### Materials

#### Translation

Tasks were translated into Mandarin using simplified Chinese characters. This format was chosen because of its high degree of accessibility, encompassing one of the primary spoken and written language forms of mainland China, Taiwan,^3^ and Singapore. Each task was initially translated into Mandarin by a bilingual researcher on the team (WGL). Tasks were then back-translated into English by a second, independent bilingual Mandarin-English speaker, blind to the original English scripts. Discrepancies between the original and back-translated English versions were identified by the research team. The process of decentering was then used (Werner & Campbell, 1970), whereby concurrent amendment of both Mandarin and English stimuli sets results in two equivalent language forms of each task. This may involve changing either language version or revising entire items. This process of back-translation and decentering allows ecological validity to be maintained in both language versions of the tasks (Brislin, 1970). Final versions of the tasks were checked by a third independent bilingual Mandarin-English speaker. This method of translation was applied to all tasks. For each task, formatting was identical in both language versions.

#### Attention bias tasks

##### Emotional Stroop

The emotional Stroop task measures attentional competition by comparing time taken to name the font color of a series of emotional words with time taken to name the font color of a series of neutral words. Increased allocation of attention to emotionally valenced words is assumed to increase their color-naming time in comparison with color-naming of neutral stimuli, giving a larger reaction time interference score, or attention bias. Stimuli comprised 80 words; 20 socially threatening, 20 physically threatening, 20 positive, and 20 neutral. Threatening word lists (both social and physical) were compiled from previous studies (MacLeod et al., 1986; Mathews & MacLeod, 1985), whereas positive and neutral word lists were compiled from the Affective Norms for English (ANEW) database (Bradley & Lang, 1999). Words were selected taking into account ecological validity in both English and Chinese cultures to ensure that words were easily understood in both language versions of the task. For example, the word “fraud” (socially threatening) was not easily understandable in Mandarin, with the concept of fraud not having been integrated into the civil law system in mainland China (Huang, 2014), and so was replaced with the word “fault.” English versions of word lists were matched for word length and frequency (Kuçera & Francis, 1967) to ensure no significant differences between lists (word length: *F* (3, 76) = 0.43, *p* = .73 frequency: *F*(3, 69) = 1.58, *p* = .20). Mandarin word lists were also matched for length (*F*(3, 76) = 1.02, *p* = .39).,^4^ ^5^

Each emotion type (socially threatening, physically threatening, positive, and neutral) involved presentation of a block of 20 words on a computer screen, with individual words colored either red, blue, yellow or green, using E-prime 2.0 (Schneider, Eschman, & Zuccolotto, 2012). Blocking is known to be a more robust method, producing larger effect sizes in emotional Stroop tasks (Holle, Neely, & Heimberg, 1997). Each block was presented four times, in a unique random order per participant, giving 16 consecutive blocks in total. Order of individual words within the array, as well as ink color assignment, was randomized separately on each new block presentation.

Participants were instructed to name the color of each word in the respective language, as quickly as possible, from left to right across each row. Time taken to color-name each entire block was recorded using a stopwatch.

##### Attention probe

The attention probe task measures attention bias by comparing the time taken to react to a neutral stimulus which replaces either a neutral or an emotionally valenced stimulus. If attention is captured by the emotionally valenced word, time taken to react to the probe in the neutral-word location will be longer than that for the emotion-word location. This reaction time difference is computed to provide the measure of attention bias. Stimuli comprised of 12 socially threatening words, 12 physically threatening words and 12 positive words, each paired with a neutral word. Word lists were compiled using the same method described above and English words were matched for length (*F*(5, 138) = 1.81, *p* = .12) and frequency (*F*(5, 120) = 1.63, *p* = .16; Kuçera & Francis, 1967). Mandarin word lists were matched for length (*F*(5, 66) = 0.67, *p* = .65). No word was used in both the emotional Stroop and the attention probe task.

Participants were instructed to focus on a central fixation cross and to identify a neutral target probe (“1” or “5”) as soon as possible but without making mistakes.^6^ Participants recorded their response by performing a button-press on a Serial Response Box. In each trial, participants were presented with a fixation cross. Emotion/neutral word pairs were presented in a random order on each side of a fixation point for 500 ms, after which the probe was presented in the location of one of the two previously presented words. Words subtended a visual angle of approximately 6° from the fixation cue.

#### Interpretation bias tasks

##### Scrambled sentence task (SST)

This task has an established sensitivity to detect interpretation biases in emotional disorders (Rude, Valdez, Odom, & Ebrahimi, 2003). It has been used in recent studies to quantify the degree to which interpretation bias explains the variance in self-reported symptoms related to different disorders, including depression (Lee, Mathews, Shergill, & Yiend, 2016) and paranoia (Savulich, Freeman, Shergill, & Yiend, 2015). The SST indicates what proportion of ambiguous information is interpreted in a positive and negative manner to give an interpretation bias score. Materials for this task were adapted from Wenzlaff and Bates (1998). Fifteen items were selected according to their suitability for translation into Mandarin and ecological validity in both languages. Each item comprised six words, five of which could be unscrambled to make a grammatically correct sentence. Sentences could be unscrambled positively or negatively depending on which words were chosen. Participants unscrambled the sentences by placing a number over the words, indicating their position in the sentence. They were instructed to use five words to form each sentence and that each sentence should be a statement not a question. Written simplified Chinese includes a small space between each character, but there is not usually a larger space between “words.” As such, to maintain congruency with the formatting of the task in English, Chinese characters were clustered in groups of no more than three characters, each forming “words,” which were then unscrambled in the same manner as English words. Instructions for the task were delivered interactively to the participant by the researcher and were therefore always given in English.

Participants unscrambled sentences under timed conditions (3 min) and high cognitive load. Before unscrambling the sentences, a 6-digit number was learned, which then had to be recalled after having unscrambled the sentences. Learning of the 6-digit number necessitated the participant recalling the number correctly on two consecutive occasions. Recall of the number took place in the language corresponding to the task version used. As each participant completed the task twice (once in Mandarin and once in English) two different 6-digit numbers were used (720185, 615239). Order of number presentation was counterbalanced between languages.^7^

##### Similarity ratings task (SRT)

The SRT measures interpretation bias through the use of emotionally ambiguous passages. Participants rate disambiguated statements related to previously seen passages for similarity. Those with a tendency towards negative interpretation of information are likely to rate negatively disambiguated statements as more similar to the original passage, in comparison with positively disambiguated statements. The task has an established sensitivity to detect interpretation biases across a wide range of disorders and trait vulnerabilities, including depression (Yiend, Lee, Tekes, et al., 2014), anxiety (Hoppitt, Mathews, Yiend, & Mackintosh, 2010), eating disorders (Yiend, Parnes, Shepherd, Roche, & Cooper, 2014) and perfectionism (Yiend, Savulich, Coughtrey, & Shafran, 2011). Materials for the SRT were adapted from Lester et al. (2011). Fifteen passages, each emotionally ambiguous in content, were selected according to suitability for translation into Mandarin and for ecological validity in both languages.^8^ Each passage was presented individually under a separate heading. Passages comprised three sentences presented sequentially , with participants pressing a button to bring up each consecutive sentence. The last word of each passage was incomplete. Participants were instructed to press the space bar as soon as they knew what the word fragment was and then to key in the first missing letter. Participants then answered a yes/no comprehension question about the passage. An example follows:

Presentation

You give a presentation during class.

People look interested and applaud at the end.

However, you feel you cannot answer the last qu-s-i-n [question].

Did you give a presentation during class? [Yes]

It is not possible to have a partially incomplete character in simplified Chinese, so in the Mandarin version of the task, the last character of the passage was missing. Characters were written onto stickers and placed on the keyboard of the testing laptop, alongside the English letter on each key. In this way, participants could select characters in the Mandarin version in the same way they could letters in the English version. Participants were asked to read the characters present on the keyboard before starting the main task to ensure familiarity with the available characters and their respective keyboard locations.

After all 15 emotionally ambiguous passages had been presented, participants were presented with test sentences for rating. They were shown the title of each passage in turn, accompanied by four separate sentences and asked to rate the similarity of each to the previously seen passage. Two of the sentences (target items) related to the ambiguous passage and were disambiguated interpretations (positive and negative) of the previous passage. The remaining two sentences (foil items) did not relate to the ambiguous passage, but were positively and negatively valenced interpretations of the emotional content, which controls for response bias. Perceived similarity of each sentence to the ambiguous passage was rated on a 4-point Likert scale, with 1–4 indicating “very different,” “fairly different,” “fairly similar,” and “very similar,” respectively. The target (positive and negative; T+ and T− respectively) and foil (positive and negative; F+ and F− respectively) sentences for the earlier example follow:

- Your presentation is successful[T+]
- Your presentation is unsuccessful[T−]
- You are generally a good writer[F+]
- You are generally a bad writer[F−]

### Procedure

All participants completed testing sessions individually in a quiet room. After giving informed consent they completed language comprehension tests prior to the main cognitive bias tasks. Three of the four cognitive bias tasks (attention probe task, emotional Stroop task, and SRT) were completed on a Fujitsu Lifebook UH572 13-inch laptop running E-Prime 2.0 (Schneider et al., 2012). The SST was completed using pen and paper. Each participant completed eight cognitive bias tasks, once in Mandarin and once in English. The order of presentation was counterbalanced using a Latin square to give 24 possible orders of presentation.

## Results

### Analysis

Data were analysed using SPSS 20, with alpha set at *p* < .05. Raw data have been deposited in the UK Data Service. Analysis first examined internal and split half reliabilities within each task independently according to language. Cronbach’s α was calculated for bias scores for all tasks as a measure of internal reliability. A value of Cronbach’s α of .7 or above is generally considered adequate for a psychological test (Kline, 1999). Next, correlations between bias scores across the respective Mandarin and English language version of each task were examined. Finally, paired samples t-tests and repeated-measures ANOVAs were used to test bias scores for any statistically significant within-group differences between different language versions of the same task.

### Emotional Stroop

Emotion interference scores were calculated for each of the three emotion types (physically threatening, socially threatening, and positive) by subtracting the time taken to name colors in the neutral condition from the time taken to name the colors in each of the emotionally valenced conditions respectively. Mean and standard deviations of interference scores are reported in Table 1. As also shown in Table 1, reliability values were good (>0.7) for both Mandarin and English versions. As shown in Table 2, correlations between Mandarin and English emotion interference scores were mostly close to zero and all were non-significant (*p* = .23–.46).

**Table 1:** Condition means (standard deviation) and reliability data for each cognitive bias task

Task	Condition mean	Chinese			English		
		Mean (SD)	Cronbach’s alpha	Split-half reliability	Mean (SD)	Cronbach’s alpha	Split-half reliability
Emotional Stroop (interference score; sec)	Physical threat	0.06 (1.51)	.47	.70	0.14 (1.28)	.60	.61
	Social threat	−0.04 (1.21)	.51	.50	0.09 (1.08)	.23	-.11
	Positive	−0.22 (1.39)	.54	.35	−0.07 (0.98)	.19	.39
	Total		.76	.78		.70	.85
Attention probe (emotion bias score; ms)	Physical threat	5.55 (46.89)	.91	.91	18.47 (54.16)	1.00	.99
	Social threat	7.42 (65.19)	.99	.99	5.31 (54.73)	.99	1.00
	Positive	24.45 (69.46)	1.00	1.00	12.25 (60.72)	.99	.99
	Total		.95	.88		.99	.95
SST (bias score)	Negative	0.16 (0.15)	.67	.58	0.18 (0.15)	.73	.67
SRT (bias score)	Positive	0.69 (0.49)	.62	.56	0.79 (0.49)	.63	.66

**Table 2:** Correlations between Mandarin and English language versions of each cognitive bias task

Task	Emotion bias type	*N*	*r*	*p*
Emotional Stroop	Physical threat	46	−.07	.32
(interference score; sec)	Social threat	46	.01	.46
	Positive	46	.11	.23
Attention probe	Physical threat	44	.06	.35
(emotion bias score; ms)	Social threat	44	.03	.42
	Positive	44	.07	.32
SST (bias score)		47	.54	<.001*
SRT (bias score)	Target	46	.65	<.001*
	Foil	46	.51	<.001*

* Correlation is significant at the 0.05 level (1-tailed)

A language (Mandarin, English) × emotion type (socially threatening, physically threatening, and positive) within-subjects ANOVA revealed no significant main effect of language (*F*(1, 45) = 0.31, *p* = .58, *η*_*p*_^*2*^ = .007) or emotion type (*F*(2, 90) = 1.81, *p* = .17, *η*_*p*_^*2*^ = .039), and no interaction effect between language and emotion type (*F*(2, 90) = 0.04, *p* = .96, *η*_*p*_^*2*^ = .001). When order of presentation of languages was included as an additional between-subjects factor there were no further significant effects. Likewise, including gender as an additional factor did not reveal any significant interactions.

### Attention probe

Two participants made over 20% inaccurate responses and their data were therefore excluded from analysis. RT outliers of less than 200 ms were excluded as anticipatory responses, as were any data points that lay more than 2.5 standard deviations above the group mean. This gave upper outlier limits of 787 ms for the task completed in Mandarin and 756 ms for the task completed in English. A total of 64 data points (6.4%) were excluded.

Emotion bias scores were calculated on the cleaned data for each emotion type (socially threatening, physically threatening, positive) by subtracting RTs when the emotion word location was probed from when the neutral word location was probed. Means and standard deviations of emotion bias scores in Mandarin and English are reported in Table 1.

As also shown in Table 1, reliability values were excellent (>0.85) for both Mandarin and English versions. As shown in Table 2, correlations between Mandarin and English emotion bias scores were close to zero and non-significant (*p* = .32–.42).

A language × emotion type within-subjects ANOVA showed no main effects of language (*F*(1, 43) = 0.00, *p* = .95, *η*_*p*_^*2*^ = .000) or emotion type (*F*(2, 86) = 1.34, *p* = .27, *η*_*p*_^*2*^ = .030) and no interaction (*F*(2, 86) = 1.13, *p* = .33, *η*_*p*_^*2*^ = .026). There were no significant order of language presentation effects, nor were there any effects of gender.

### SST

Negative bias scores were calculated as a fraction, with the denominator being the total number of items attempted (including incorrect or unfinished items). The numerator included only those items completed correctly, using five words in a grammatically correct order with a negative interpretation.

As shown in Table 1, reliability values were adequate (0.58–0.73) for both Mandarin and English versions. Mandarin and English negative bias scores showed a statistically significant one-tailed positive correlation, *r*(45) = .54, *p* < .001.

A paired samples t-test indicated that there was no statistically significant difference between negative bias scores in Mandarin and English versions of the task, *t*(46) = 1.02, *p* = .31, *d* = 0.30). There were no effects of order of language presentation or gender on negative bias scores.

### SRT

Bias scores were calculated for Mandarin and English versions of the task. Bias scores were calculated for both target and foil sentences (separately) by subtracting similarity rating scores for sentences framed negatively (negative targets/ negative foils) from those framed positively (positive targets/ positive foils), thus providing an index in which a higher score indicated a more positive bias.

As shown in Table 1, reliability values were adequate (0.56–0.66) for both Mandarin and English versions. There was a statistically significant one-tailed positive correlation between bias scores in Mandarin and English for target sentences, *r*(44) = .65, *p* < .001, and for foil sentences, *r*(44) = .51, *p* < .001 (see Table 2).

Paired samples t-tests were carried out on positive bias scores in Mandarin and English. There was no significant difference between positive bias scores in the two versions of the task either for target sentences (*t*(45) = 1.68, *p* = .10, *d* = 0.50) or for foil sentences (*t*(45) = 0.44, *p* = .66, *d* = 0.13), suggesting similar performance across both versions. There were no order effects of language presentation or gender on positive bias scores.

## Discussion

Performance on measures of interpretation bias was broadly equivalent across languages, with good correlations between bias scores. However, performance on measures of attention bias showed little evidence of measurement equivalence across language, with correlations that were close to zero in most cases. Both kinds of tasks showed adequate to good internal reliability across both language versions. Our results on measures of interpretation were clear, showing measurement equivalence across language versions as well as adequate reliability and we therefore conclude that these measures are suitable for future cross-cultural research. Further work should build on the current findings by establishing construct equivalence, for example by comparing factor structures across native samples.

In contrast, our findings on attention bias tasks did not demonstrate measurement equivalence and require more in depth discussion. The attention task data raise important issues about the consequences of using bilingual samples to assess task measurement equivalence. Bilinguals have fundamental differences in their first and second language skills (Dornic, 1979; Winskel, 2013). For example, the first language learned tends to carry more “emotional” meaning than subsequent languages learned (Altarriba, 2008; Pavlenko, 2008), as we noted earlier. One important consequence arising from this for the present study is that emotional attention and interpretation bias effects may have been relatively intensified in the dominant language, and relatively weakened in the second language. Although this could explain a lack of measurement equivalence across language versions of the tasks, one might have expected it to affect all tasks, rather than attention tasks only. Of course, it is possible that this was the case, but that the inherently larger effects typically found on interpretation bias tasks were able to survive through the second language, whereas the small effects found in attention tasks were not. In addition to the longer processing time allowed by interpretation bias tasks, it is also possible that they include more social content than attention bias tasks, increasing their “emotional weight.” This in turn could explain the larger effect sizes seen in the interpretation bias tasks.

There is a further possible explanation for the pattern of results on attention bias tasks, related to cultural priming. Language can be used as a prime for cultural values (Oyserman & Lee, 2008) and the degree of priming will be determined by a person’s level of Bicultural Identity Integration (BII; Benet-Martinez & Haritatos, 2005). The pattern of results found on the attentional tasks namely, low within-participant correlations between English and Mandarin versions, but no significant differences between means, would be consistent with this. The sample may have included some participants with high BII who demonstrated assimilation and some low BII participants who showed a contrast effect. However, as before, this phenomenon should have affected both attention and interpretation bias tasks similarly and we therefore suggest that other explanations are more likely.

The explanation that we favor for the pattern of results seen across attention and interpretation tasks is one based around language effects, and is again a consequence of using a bilingual sample. We suggest that there is an intriguing possibility that inherent differences in speed of access to semantic content across languages may explain the pattern of results observed. Experimental research in bilinguals shows that accessing (non-emotional) semantic content takes longer in the second language (Favreau & Segalowitz, 1983), than in the dominant language (Dornic, 1979), even though participants can achieve the same level of language comprehension in both languages when reading. In our study most participants were native Mandarin speakers, with English being their second language. It is therefore possible that access to the meanings (irrespective of emotional content) of the English words was compromised during attentional tasks because processing time was inherently limited by the task design. Unlike interpretation tasks, attention bias tasks require participants to speedily process semantic meaning, in order for bias effects to be observed (Williams, Mathews, & MacLeod, 1996). Slower speed of semantic processing in the weaker, second, versus the dominant, native language (Favreau & Segalowitz, 1983) would compromise participants’ access to semantic content during attention tasks when presented in their non-native language. For example, the attention probe task requires a short duration (500 ms) for stimulus presentation. When presented in the second language, this may have been too short to allow participants sufficient time to encode the entire semantic content of both words in each pair. This in turn would mean that attention bias interference effects, driven as they are by the encoded meaning of each word, were not detectable during the time window that was sampled by the task. As the interpretation bias tasks did not restrict stimulus processing time in this way, but instead were self-paced, they would not have been subject to the same problem.

One possible method of testing, and combatting, the above putative phenomenon would be to increase the length of time that stimuli are presented for during the attention tasks. However, there is no guarantee that a uniform increase in duration would yield uniform increases in access to semantic content across participants. Language proficiency is thought to mediate automatic arousal for emotional words (Winskel, 2013), suggesting that the optimum window of stimulus presentation duration would vary within bilinguals themselves. If correct, the implications of the preceding discussion for developing culturally appropriate tests is that validation of time-critical tasks should only take place in the native language. An alternative route to conduct culturally appropriate development of attention bias tasks would be to abandon verbal stimuli altogether and instead use tasks involving pictorial stimuli or culturally appropriate facial expressions of emotion.

To the best of our knowledge, this is the first instance in which measures have been developed to investigate cognitive biases in different cultures. Our attempts at development of parallel materials measuring bias in Chinese (Mandarin) and English is the first step towards the investigation of cross-cultural differences in biased cognition. The causal nature of interpretation bias in the vulnerability to psychopathology (Yiend & Mathews, 2004) and its role in maintaining psychopathology (Savulich, Shergill, & Yiend, 2012) heightens the importance of its investigation across different cultures. Earlier we illustrated how existing cross-cultural data suggesting that interdependent, self-criticizing Easterners might be expected to show attenuated positive cognitive biases, and be more susceptible to negative biases, compared to independent, self-enhancing Westerners. Further supporting this view, we discussed data showing cultural differences in anxiety and depression relating to self-construals. However, in a recent paper, Curhan and colleagues (2014) present a view which might lead to the opposite prediction. They postulate that there may be cultural differences in the way that negative feelings are understood by individuals. Westerners, they suggest, interpret negative emotions as a personal failing, shouldering the associated burden of the failure, whereas Easterners are more likely to believe that negative emotions are a result of natural cycles and exist in the relationships between people, thus avoiding personal burden. In this view, Easterners would be *less* vulnerable than Westerners to the adverse consequences of experiencing negative affect, suggesting a corresponding pattern of reduced negative and enhanced positive biases in Eastern participants. Clearly the predictions from the above position are opposite to those arising from the literature on self enhancement, anxiety and depression and only further research will be able to delineate the true pattern of cultural differences in biased processing. At present we can only speculate, but the differing implications from the literature serve to highlight the need for further investigation of cross-cultural differences in biased cognition.

There are also important translational reasons why cultural differences in biased cognition need to be fully understood, namely the implications of these findings for an individual’s vulnerability to psychopathology. If biased cognition varies across cultures, then it is likely that psychological treatments targeting cognitive mechanisms may be differentially effective, and may need to be more culturally specific than has, to date, been assumed. Psychotherapy that attempts to influence maladaptive cognitive processes, such as cognitive behavioral therapy (CBT), has been shown to be efficacious in different clinical populations (Hofmann, Asnaani, Vonk, Sawyer, & Fang, 2012; Nathan & Gorman, 1998), but the majority of research has been carried out in Western populations. Although Western theories of psychotherapy have been shown to be applicable to the Chinese population (Hodges & Oei, 2007; Liu & Leung, 2010), it has been argued that changes to the structure of CBT need to be made in order to better accommodate values held by Chinese individuals (Hodges & Oei, 2007). It is currently unknown whether some techniques that are currently used in psychotherapy may be less effective in Eastern populations, and better understanding of possible differences in the underlying cognitive mechanisms driving psychopathology, such as biased processing, could throw light on this question. Determining the typical patterns of attention and interpretation bias demonstrated in collectivist cultures will help inform the implementation of psychotherapeutic treatments, potentially increasing the efficacy of treatment in Eastern cultures.

The present study has various limitations. We did not include measures of individual differences that may moderate performance on these measures of cognitive bias, such as self-construal, trait anxiety, or trait depression. Our focus here was exclusively on test development and equivalence across different language versions. It will be important for future work to examine moderators related to individual differences. Often one shortcoming, especially where specialized populations are concerned, is the small size of the participant cohort; however, this was not the case in the current experiment. Although data from nine participants had to be excluded due to English language comprehension inadequacy, an ample-sized cohort of 47 individuals remained. As discussed at length above, possible differences in second language ability was a further limitation of the present work. Cognitive tasks are rarely “process pure,” and this limitation is particularly true of the SST task, which may reflect selective attentional capture (e.g., if participants' unscrambled sentence choice was, in part, driven by greater salience of a valenced word) as well as interpretation. Gathering convergent evidence from additional tasks of the same putative cognitive process, as we did here, is one way to mitigate the methodological constraints imposed by individual tasks.

The development of materials suitable for measurement of cognitive biases in Eastern cultures is an important novel progression for the field. The interpretation bias tasks presented here showed equivalence across language versions, had adequate reliability and were suitable for future cross-cultural research. In contrast, attention tasks lacked sufficient evidence of equivalence, suggesting that cross-cultural investigation should instead use nonverbal stimuli such as pictures or facial expressions of emotion. These data mark the starting point for future research investigating East-West differences in biased cognition. Using cross culturally valid materials, it will be possible to explore whether patterns of cognitive bias in both healthy and clinical populations shown in Eastern cultures are comparable to those already documented in Western cultures, with corresponding implications for psychological treatments for mental health disorders.

## Footnotes

1 We also provide all task materials and instructions online in the Open Science Framework (attention probe task: https://osf.io/upj74/. Emotional Stroop task: https://osf.io/bn8km/. SST: https://osf.io/7vjmw/. SRT: https://osf.io/7pk9v/); raw data can be found in the UK Data Service (http://reshare.ukdataservice.ac.uk/852440/).

2 English: http://englishteststore.net/index.php?option=com_content&view=article&id=2974:English-Intermediate-Reading-Comprehension-Test-001&catid=133:english-reading-tests&Itemid=382; Mandarin: http://www.esl-languages.com/en/study-abroad/online-tests/chinese-test/index.htm

3 Mandarin is spoken in Taiwan, as in mainland China, but traditional Chinese characters are used rather than simplified Chinese characters.

4 All words were matched for frequency using English-language norms, but it is likely that words have similar frequencies across cultures. Chinese word frequencies were checked using Cai and Brysbaert (2010). This is the only database which allows the frequency of multiple-character clusters to be checked.

5 Word lists and written instructions to participants for the emotional Stroop task, in both languages, can be found on the Open Science Framework (https://osf.io/bn8km/)

6 Full instructions and task materials for the attention probe task can be found on the Open Science Framework (https://osf.io/upj74/)

7 Full SST task materials can be found on the Open Science Framework (https://osf.io/7vjmw/)

8 Full SRT task materials can be found on the Open Science Framework (https://osf.io/7pk9v/)
